# Finding a Balance in the Vaginal Microbiome: How Do We Treat and Prevent the Occurrence of Bacterial Vaginosis?

**DOI:** 10.3390/antibiotics10060719

**Published:** 2021-06-15

**Authors:** Rebecca Jane Joseph, Hooi-Leng Ser, Yi-He Kuai, Loh Teng-Hern Tan, Valliammai Jayanthi Thirunavuk Arasoo, Vengadesh Letchumanan, Lijing Wang, Priyia Pusparajah, Bey-Hing Goh, Nurul-Syakima Ab Mutalib, Kok-Gan Chan, Learn-Han Lee

**Affiliations:** 1 Novel Bacteria and Drug Discovery Research Group (NBDD), Microbes and Bioresource Research Strength (MBRS), Jeffrey Cheah School of Medicine and Health Sciences, Monash University Malaysia, Bandar Sunway 47500, Malaysia; Rebecca.Jane@monash.edu (R.J.J.); ser.hooileng@monash.edu (H.-L.S.); Yi-He.Kuai@monash.edu (Y.-H.K.); loh.teng.hern@monash.edu (L.T.-H.T.); Vengadesh.Letchumanan1@monash.edu (V.L.); priyia.pusparajah@monash.edu (P.P.); syakima@ppukm.ukm.edu.my (N.-S.A.M.); 2 Clinical School Johor Bahru, Jeffrey Cheah School of Medicine and Health Sciences, Monash University Malaysia, Johor Bahru 80100, Malaysia; t.jayanthi@monash.edu; 3 Vascular Biology Research Institute, Guangdong Pharmaceutical University, Guangzhou 510006, China; wanglijing62@163.com; 4 Biofunctional Molecule Exploratory Research Group (BMEX), School of Pharmacy, Monash University Malaysia, Bandar Sunway 47500, Malaysia; goh.bey.hing@monash.edu; 5 College of Pharmaceutical Sciences, Zhejiang University, Hangzhou 310058, China; 6 UKM Medical Molecular Biology Institute (UMBI), UKM Medical Centre, Universiti Kebangsaan Malaysia, Kuala Lumpur 56000, Malaysia; 7 Division of Genetics and Molecular Biology, Institute of Biological Sciences, Faculty of Science, University of Malaya, Kuala Lumpur 50603, Malaysia; 8 International Genome Centre, Jiangsu University, Zhenjiang 212013, China

**Keywords:** bacterial vaginosis, microbiome, probiotics, *Lactobacillus*, *Gardnerella*, VMT

## Abstract

Bacterial vaginosis (BV) has been reported in one-third of women worldwide at different life stages, due to the complex balance in the ecology of the vaginal microbiota. It is a common cause of abnormal vaginal discharge and is associated with other health issues. Since the first description of anaerobic microbes associated with BV like *Gardnerella vaginalis* in the 1950s, researchers have stepped up the game by incorporating advanced molecular tools to monitor and evaluate the extent of dysbiosis within the vaginal microbiome, particularly on how specific microbial population changes compared to a healthy state. Moreover, treatment failure and BV recurrence rate remain high despite the standard antibiotic treatment. Consequently, researchers have been probing into alternative or adjunct treatments, including probiotics or even vaginal microbiota transplants, to ensure successful treatment outcomes and reduce the colonization by pathogenic microbes of the female reproductive tract. The current review summarizes the latest findings in probiotics use for BV and explores the potential of vaginal microbiota transplants in restoring vaginal health.

## 1. Introduction

The colonization of the vaginal microbiome begins to occur at birth, just like the gut or skin microbiome, and is subject to variation depending on delivery mode (i.e., vaginal birth or cesarean section) [1,2,3]. Just as the morphology and physiology of the vagina change throughout a woman’s life, the vaginal microbiome is also affected by factors such as the onset of puberty and hormonal changes during the menstrual cycle, menopause as well as pregnancy [4,5,6]. Therefore, it is very important to bear in mind that just as the vaginal microbiota can affect the host’s reproductive physiology, the microbial composition can also be influenced by host physiology.

Being the most common cause behind vaginal discharge with a foul odor, bacterial vaginosis (BV) can occur when there is an imbalance in the vaginal microbiome (i.e., reduction in *Lactobacillus* spp. abundance) and overgrowth of certain microbial populations(s) in the vagina [7,8]. BV-affected women may also encounter itch/burning sensation and discomfort around the intimate area [7,9]. While many individuals may not display symptoms during BV, one of the main reasons contributing to the poor health-seeking behavior of vaginal discharge is shame and fear of judgment by others, which accentuates the need to increase the awareness of BV [10]. The current review aims to provide an overview of BV, covering several aspects, including diagnostic methods and guidelines, before venturing into exciting new developments in the treatment and management plan of the problematic bacterial overgrowth in the vagina.

## 2. The Development of the Vaginal Microbiome: How Does It Change throughout a Woman’s Life?

During the first few weeks of life, parts of female reproductive tract (FRT) like the vagina and vulva, are affected by residual maternal estrogens [11]. As the vagina mucosa contains a rich glycogen supply, it has been proposed that lactic acid bacteria like *Lactobacillus* species quickly colonize it within the first 24 h of birth [4,12]. As the glycogen content drops in the vagina, it is presumed that the vaginal pH becomes neutral or alkaline due to the relative deficiencies of lactic acid-producing microbes [13,14,15]. Most of the studies reporting vaginal microbiome relied on conventional cultivation and microscopic examinations [15,16,17]. In the early 19th century, several studies attempted to explore the normal vaginal flora in children by cultivation methods before identification via microscopic observation and Gram staining [16,18,19]. With the improvement and introduction of enrichment culture media, a study by Gerstner and team used different sets of media to study aerobic and anaerobic microbes from vaginal swab samples (obtained during speculum examination) of prepubertal children (age range: 3 months to 16 years old; mean age: 9 years old) [15]. Gerstner and team showed that *Lactobacillus* made up the largest population of aerotolerant anaerobes (or microaerophilic) in asymptomatic children (i.e., children attended to the clinic for reasons other than vaginal infections/symptoms). Keeping in mind that identification via cultivation methods leaves out some fastidious or difficult-to-culture microbes, there were some doubts regarding the findings on the vaginal microbiome in children [20,21,22,23]. Later in 2015, a thorough study performed an analysis of the vaginal microbiome in pre-menarche adolescent girls using next-generation sequencing technology [24]. Hickey et al. performed a three-year longitudinal study to examine how vaginal microbiome changes as children transition through menarche. Targeting the V1 to V3 region of the 16S rRNA gene, the team performed metagenomic analyses using Roche 454 platform and observed that *Lactobacillales* (predominantly *Lactobacillus* spp.) represented more than half of the community in 80.5% of participants (*n* = 25). In some cases, *Streptococcus* spp. was also detected. Furthermore, nearly all participants who reached menarche (*n* = 21) displayed *Lactobacillus* dominance before or shortly after menarche. In respect of species composition and temporal dynamics, some participants showed considerable deviations. For example, one participant displayed a gradual shift of *Lactobacillus iners* dominance to *Lactobacillus crispatus* at different visits, while another participant displayed a transition of aerobic microbes to *Lactobacillus jensenii*, *Lactobacillus gasseri*, and *Bifidobacterium* spp. at menarche. Nonetheless, the team was left puzzled after discovering that several vaginal samples had pH well above 4.5, even though there were high proportions of lactic acid bacteria. While the finding warrants further investigation to study the transition of vaginal microbiome, the study concluded that vaginal microbiota of girls begins to resemble those adults even before the onset of menarche.

Generally, a healthy adult vaginal microbiome predominantly consists of *Lactobacillus* spp. and other microbes at lower abundances such as *Peptostreptococcus* spp., *Bacteroides* spp. and *Enterobacteriaceae* [25,26,27,28,29]. Apart from creating an acidic environment that favors their growth, most of the *Lactobacillus* strains can generate an appreciable number of antimicrobial-like compounds, bacteriocin, or hydrogen peroxide, which is toxic to a range of potentially harmful microbes and inhibits their growth [30,31]. Apart from that, certain *Lactobacillus* spp. are also capable of regulating the host’s immune response to purge or keep pathogens out of the FRT [28]. *Lactobacillus* spp. has been shown to exhibit inhibitory effects on pathogens by resource competition, representing another key mechanism in maintaining the colonization of the vagina [32,33,34,35]. Despite that, it is still impossible to pinpoint precisely which species or groups of *Lactobacillus* spp. are solely responsible for maintaining the eubiosis within the vaginal microbiome [36]. Multiple types of *Lactobacillus* spp. are numerically dominant at different time points within the same individual [37]. A study by Ravel et al. in 2011 described five types of vaginal bacterial communities among reproductive-age women, called Community State Types (CST). It is totally normal for women to experience change from one CST to another within a short period of time. Each CST has got a predominant *Lactobacillus* species: (a) Type I: *L. crispatus,* (b) Type II: *L. gasseri*, (c) Type III: *L. iners*, and (d) Type V: *L. jensenii* [36]. On the contrary, CST IV lacks *Lactobacillus* spp. and is generally dominated by a diverse range of facultative or strict anaerobic bacteria like *Megasphera, Prevotella, Gardenella*, and *Sneathia*. While women may appear to be apparently healthy with CST IV, this CST is often associated with a high Nugent score and bacterial vaginosis (BV) [36,38,39]. While the actual reasons behind these differences are still being explored, some studies speculated that these are due to racial variation as well as host genetic differences [29,36,40,41].

Just as host behaviors such as hygiene and sexual practice directly impact microbial composition in the vagina, the selection of non-hormonal birth control methods such as the use of an intrauterine device (IUD) may disrupt the balance of the vaginal microbiome, increasing infection risk [42,43,44,45,46]. Numerous studies have suggested the hormonal contraceptives like medroxyprogesterone acetate disrupt the vaginal epithelial layer and alter immune response, leading to alterations in the vaginal microbiome and possibly increases host susceptibility to HIV-1 [47,48,49]. Be it as may, increasing evidence is piling up that estrogen plays a critical role in the maintenance of the vaginal microbiome. Among physiological alterations that occur during pregnancy to adapt to pregnancy-specific physiological processes in mother and fetus, one of the important physiological changes would be increased estrogen level, which directly impacts the vaginal microbiome [50,51,52,53]. The rising level of estrogen promotes glycogen synthesis within the vaginal epithelium, which in turn selectively enriches the growth of *Lactobacillus* spp. [54]. Even though *Lactobacillus* does not produce alpha-amylase, the human host enzymes and other bacterial pathways metabolize glycogen into compounds (e.g., maltose, maltotriose, maltopentaose, and maltodextrins) that are readily available to support the growth of these bacteria [55]. *Lactobacillus* spp. metabolizes these smaller compounds, which produces lactic acid, resulting in an acidic environment that gives them an upper hand in colonizing the vagina at the cost of other microbes [56,57,58,59,60]. Romero et al. compared the vaginal microbiome of healthy, non-pregnant women to pregnant women who delivered at term [61]. They discovered that the vaginal microbiota during pregnancy display higher stability compared to the non-pregnant state. Even though the transition of *Lactobacillus*-dominated CST occurs during pregnancy, but seldom to CST-IV. Another study in 2019 by Serrano et al. noticed a significantly lower alpha diversities (*p* < 0.01) of the vaginal microbiomes of pregnant women to case-matched (i.e., race, gestational age, and household income) non-pregnant women [62]. In contrast to pregnancy, women in the postmenopausal phase (typically around 50 years old) would experience major alterations in reproductive hormones, including decreased estrogen and increased follicle-stimulating hormone levels [63,64,65]. Mirmonsef et al. spotted a significant reduction of free glycogen in post-menopausal women compared to pre-menopausal women (median 0.002 vs. 0.065 µg/µL, respectively; *p* = 0.03), which correlated positively with *Lactobacillus* level in the vagina [4,66]. Subsequently, in 2019, Gliniewicz et al. compared the vaginal microbiomes of pre- and postmenopausal women, including those on hormone replacement therapy (HRT) [67]. By quantitating the 16s rRNA gene copies, the team observed that postmenopausal women receiving HRT showed comparable bacterial numbers with pre-menopausal women. However, postmenopausal women who were not on HRT displayed around 10-fold lesser bacterial count than the other two groups (i.e., pre-menopausal and postmenopausal women on HRT; *p* < 0.05). Along with a reduced abundance of *Lactobacillus* spp., many studies reported an increase in the abundance of anaerobes in postmenopausal women (e.g., *Bacteroides*, *Mobiluncus*) along with vaginosis-associated bacteria like *Gardnerella vaginalis* [68,69,70]. Shardell et al. performed a two-year cohort study (*n* = 750) comprised of pre-, peri-, and post-menopausal women [69]. Nearly half of the postmenopausal women (49.7%, 356/716) were found to exhibit a vaginal microbiota community with low-*Lactobacillus* spp. Furthermore, those with low-*Lactobacillus* spp. reported to have a higher occurrence of decreased libido (OR = 1.79, 95% CI = 1.04–3.12) and vaginal dryness (OR = 1.61, 95% CI = 0.89–2.90) compared to their counterparts with high-*Lactobacillus* spp. communities.

The vaginal microbiota is a unique and complex environment that can alter in response to a plethora of endogenous and exogenous factors. While there are several articles discussing ethnicity and genetic differences contribute to interindividual variations in the vaginal microbiome, it is reasonable to mention that there is still much to be explored in understanding the temporal dynamics and signatures of the vaginal microbiome in women throughout life [29,36,41].

## 3. Epidemiology of Bacterial Vaginosis (BV)

Researchers are still debating whether BV should be considered as a sexually transmitted infection, given that the chance of getting BV increases with sexual activity (multiple sex partners), vaginal douching, smoking, presence of the IUD, and antibiotic usage [71,72]. In general, the global economic burden of treating symptomatic BV is estimated to be US $4.8 billion annually, yet the prevalence of BV varies in different regions [73,74]. An earlier systematic review by Kenyon et al. in 2013 gathered information on the prevalence of BV from a total of 86 surveys conducted from different parts of the world [75]. Interestingly, while a general observation showed that parts of Africa reflected the highest prevalence of BV and lowest in most regions in Asia and Europe, there are still some populations that displayed an opposite pattern—with some areas in Africa with very low BV prevalence and some Asian and European populations with high BV rates. The team also discussed another rather fascinating point, whereby the prevalence within a country can vary by ethnic group.

In the United States, Allsworth and Peipert collected a comprehensive report from 2001 to 2004 to study the prevalence and correlations of BV among women aged between 14 to 49 years old (Table 1) [76]. The study showed that approximately one-third of the women enrolled in the National Health and Nutrition Examination Survey were tested positive for BV; among this population, those between the ages of 14 and 19 years had a lower prevalence (23.3%) compared to those aged 20 years old and above (with prevalence between 28–31%). Koumans et al. also reported similar findings in 2007 [71]. Likewise, Allsworth and Peipert (2007) mentioned that the prevalence of BV varied with age, race or ethnicity, education, and poverty Allsworth and Peipert [76]. For instance, Black, non-Hispanic, and Mexican-American women in the United States displayed a higher likelihood of bacterial vaginosis than white, non-Hispanic women after adjustment for other sociodemographic characteristics. Panning over to Mexico, a cross-sectional study conducted in Cuernavaca City recruited a group of 405 sexually active women attended to the City hospital to determine the prevalence in the city; they noted that 67/405 (16.5%) of the population displayed clinical symptoms consistent with BV, along with a commonly reported sign of having yellow secretions [77]. On the other hand, researchers from Grenada (which is a small island country in the Caribbean Sea) observed an increase of BV in trend from 16.1% in 2009 to 21.4% in 2011 among Grenadian women of reproductive age (age range: 15–49 years old) [78]. Over a three-year study period, the team discovered similar findings as presented by Allsworth and Peipert (2007), whereby those within the 20–29 years old age group were the largest percentage of infection (43.6%) in their study population.

A study that recruited participants between December 1997–June 1998 reported the BV prevalence in Peru as 30.6% using either Nugent’s or Amsel’s criteria [79]. Several years later, another team reported that 26.6% of the women (*n* = 207/779) were positive for BV when examined using a series of laboratory tests [80]. After collecting data from interviews, the group discovered 32 cases of BV reported among 107 women without sexual experience (based on self-report), which is equivalent to 15.5% of total BV cases (*n* = 207). Aside from that, it was revealed via an unadjusted analysis that the prevalence of BV correlates positively with sexual behaviors and history (e.g., sex workers, number of partners in the past year), but not variables such as being sexually experienced and mean number of unprotected sexual encounters in the past three months. As pointed out by the authors, there was a lack of information to address the association between BV and socioeconomic status when they published their work. In fact, only one study in the United Kingdom suggested an increased prevalence of BV among those with low socioeconomic status, especially when they observed a higher number of women who were living in a deprived area tend to seek diagnosis at a genitourinary clinic in Sheffield [81].

A study in Delhi, India recruited 237 women living in an urban slum, urban middle class, or rural community [84]. The overall BV prevalence was described as 32.8% based on laboratory examination. When comparing sociodemographic profiles of the study population, the highest prevalence of BV was observed in the urban slum (38.6%), followed by the rural community (28.8%) and urban middle-class community (25.4%). In contrast to other studies, the team did not notice any association between BV prevalence and age; specifically, almost no difference in prevalence was seen in women aged between 15–24 years old and those above 25 years old. Then again, Uma et al. investigated the prevalence of BV among women of childbearing age and low socioeconomic status [83]. Out of 487 women enrolled in the screening program in Chennai, India, 24.6% of them were diagnosed to have BV. Consistent with other studies, those aged above 25 years old displayed a significant association with BV, and a higher percentage of married women were shown to have BV compared to those unmarried (28% versus 12%, *p* = 0.085). When these results were compared to a previous study conducted in Chennai, the team noted that the prevalence of BV in the general population was lower (24.6%, *n* = 120/487) than the prevalence found among the female sex workers (45%, *n* = 260/582) [83,85]. Nevertheless, these results do not mean that only those in high-risk sexual behavior are associated with BV, albeit clinicians should be aware or suspect BV in women who presented vaginal odor and/or discharge. A six-month study in the neighboring country of India Nepal, published in 2018 has shown that the overall prevalence of BV was 24.4% among non-pregnant women, with the highest prevalence recorded among those aged between 30 to 40 years old (8.8%) [87].

Looking at the Africa continent, numerous groups were studying the prevalence of BV. In Ethiopia, Bitew et al. conducted a hospital-based cross-sectional study in the capital city, Addis Ababa [89]. Like studies from other regions, younger women aged 15 to 24 years old had a somewhat lower prevalence (41.5%) of BV as compared to those above 25 years old (47.8–60.0%). Besides that, among the 212 non-pregnant participants who attended a gynecology clinic in Nigeria, 85 participants (40.1%) were positive for BV, with most of them aged between 25–34 years old (*n* = 50, 58.8%) [91]. Although there are no measured parameters that can explain the high BV prevalence and racial differences compared to studies from other African countries, the authors suggested that the observation may be due to socioeconomic factors. Even so, a meta-analysis performed by Torrone et al. in 2018 commented on the high heterogeneity in studies reporting BV prevalence among women aged 25–49 years old in the Africa region [95]. Among those aged between 15–24 years old, the summary estimates for BV prevalence were 49.5% for higher-risk populations in Eastern Africa, 35.2% for South African clinic/community-based populations, and 42.1% for Southern/Eastern African clinics- or community-based studies.

On top of studying women of reproductive age, some studies are focusing on the prevalence of BV in pregnant women, given that many clinicians observed that BV is associated with preterm birth and other complications in pregnancy [92,93,96]. In the United Kingdom, Akinbiyi et al. reported that almost 90% of the BV cases among asymptomatic pregnant women were 21–30 years old [82]. Additionally, similar findings were also discovered by a team in Nigeria, in which a high prevalence (17.5%) was observed among pregnant women aged between 20–24 years old [90]. After analyzing the vaginal swabs and epidemiological data collected from 400 pregnant women with complaints of abnormal vaginal discharge, it was found out that the prevalence of BV among this selected population was 17.3%. The team further discussed that multigravidity, lack of western education, and unemployment were linked with increased BV prevalence. By the same token, another group of researchers from Ethiopia marked that factors such as multiple sexual partners (OR: 8.6; 95% CI: 2.5, 29) and previous history of spontaneous abortion (OR: 5.9; 95% CI: 1.5, 23) significantly correlated with BV prevalence in their study which randomly recruited 57 symptomatic and 195 asymptomatic pregnant women (age range: 18–40 years old) visiting obstetrics and gynecology clinic from November 2011 to April 2012 [88]. While the overall prevalence of BV reported being 19.4%, the team further analyzed the information by subcategorizing symptomatic and asymptomatic pregnant women and found the prevalence to be 31.6% and 15.9%, respectively. One of the key findings that should be made wary is that more than half of the BV-positive pregnant women were asymptomatic (63.3%). Even though some studies argued that the treatment of BV using antibiotics does not reduce or change preterm birth risk, the screening and management plans of BV among pregnant women are still perceived as a controversial issue [97,98,99,100].

Looking at BV prevalence among postmenopausal women, it is rather unexpected as this condition rarely occurs, even though they typically display a reduction in *Lactobacillus* colonization of the vagina. An earlier study by Cauci et al. in 2002 recruited non-pregnant postmenopausal women aged between 40–79 years old in Italy and reported an overall BV prevalence for all postmenopausal women as 6.0% [94]. A subgroup analysis reported that postmenopausal women receiving HRT had slightly lower BV prevalence (5.4%) than those without HRT), but the differences were not statistically significant (*p* = 0.609). Another study in India showed a higher BV prevalence compared to a study by Cauci et al., with an overall prevalence of 24.4% (*n* = 51/209, age range: 42–64 years old) [86]. Furthermore, BV was significantly high (56.86%) in postmenopausal women aged more than 55 years old compared to women aged 55 years old and lesser (*p* = 0.018). To date, few studies have noted variations in the prevalence of BV among postmenopausal women, possibly due to standard diagnostic criteria as they are more relevant to premenopausal women [86,94,101,102].

## 4. Pathogenesis of BV and the Identification of Causative Agents

BV occurs due to microbial dysbiosis, presenting a highly diverse vagina microbiome (including *Gardnerella* spp. and *Atopobium* spp.) as opposed to the healthy form, which is mainly dominated by *Lactobacillus* spp. The predominant symptom is the presence of grayish-white thin homogenous discharge with an unpleasant odor or “fishy smell,” which is more apparent during menses or after sexual intercourse due to the increased production of amines by anaerobic bacteria. The invasion of normal, healthy microflora by pathogens disrupt the host physiology via multiple routes, including depleting nutrients essential for the growth of other residents within the vagina, destroying the vaginal barrier via hydrolytic enzymes (e.g., sialidase and prolidase), and promoting the release of pro-inflammatory chemokines and cytokines (IL-6, IL-8, IL-1α, IL-1β, TNF-α) [103,104]. With the depletion of *Lactobacillus* spp., the vaginal pH fails to be maintained at the normal range (i.e., 3.8–4.5), which successively creates a cascade of undesirable events in the host, including persistent infection potentially caused by a mixture of difficult-to-treat pathogens, especially when some of them possess the ability to form biofilms [104,105,106,107,108].

Along with developing diagnostic tools and treatment plans for BV, multiple attempts can be seen throughout these years to monitor changes in the microbial composition while identifying potential causative agent(s) [89]. Recent advancements in molecular tools greatly accentuated their position as a better tool with higher resolution power as compared to traditional cultivation methods and biochemical tests, which may be difficult to be applied to fastidious organisms as well as to differentiate microbes at the species-strain level [109,110,111,112,113,114]. Several reviews have been published over the past few decades, summarizing the tools available for studying microbial composition and vaginal microbiome transition during BV [111,115,116]. One of the infamous bacteria that is highly implicated in the literature associated with BV is *G. vaginalis*. The first description of this Gram-negative facultative anaerobe was made in 1953 by Leopold as a novel microbe from patients with cervicitis and prostatitis, which later demonstrated by two researchers—Gardner and Charles D. Dukes to be associated with BV [117,118,119]. Previously, scientists have been contemplating the causative roles of *G. vaginalis* in BV as this microbe exists in women without BV, yet Janulaitiene et al. found that only particular clades of *G. vaginalis* are pathogenic, which might be due to the presence of gene coding for sialidase [120]. As we know, the first step of invasion is adherence to the host cell. As demonstrated by Patterson and team, the virulence of *G. vaginalis* can be attributed to several features: (a) the production of a cytolysin known as vaginolysin, which targets human cell specifically and activates the cell death pathways via binding to the complement regulatory molecule CD59, (b) strong adherence to host cells which avoid clearance by the host, and (c) ability to form biofilms [121]. On top of its capability in producing hydrolytic enzyme-like sialidase, which damages vaginal mucosal surfaces and thus increases the level of pro-inflammatory cytokines, *G. vaginalis* has the propensity to form dense biofilms, allowing it to persist in the environment and serve as a scaffold that supports the growth of other opportunistic pathogens [103,121,122,123,124,125,126]. The formation of a “new community” within the biofilm subsequently competes with *Lactobacillus* spp. as those living within the biofilms of *G. vaginalis* have a higher tolerance against lactic acid and hydrogen peroxide produced by *Lactobacillus* spp.; this increases their survival chances and failure to purge these opportunistic pathogens from the environment will eventually tilt the balance of the entire vaginal ecosystem [127]. Many efforts have been poured into the investigation of *G. vaginalis* biofilm, allowing identification of other opportunistic bacteria such as *Mobiluncus* spp., *Atopobium vaginae*, *Prevotella bivia*, *Mycoplasma hominis*, *Peptostreptococcus* spp., *Porphyromonas* spp., *Sneathia* spp., *Ureaplasma urealyticum*, *Leptotrichia* spp., *Candidatus* Lachnocurva vaginae (formerly known as BVAB1), *Mageeibacillus indolicus* (formerly known as BVAB3) and BV-associated bacterium type 2 (BVAB2) [103,127,128,129,130,131,132].

Having said that, members of the biofilm community often work hand in hand to ensure they thrive in the vaginal ecosystem. *P. bivia* is another commonly detected microbe found in vaginal swabs of BV patients [133,134,135,136,137]. Working adjacent with the action of *G. vaginalis*, *Prevotella* spp. including *P. bivia* and *Prevotella disiens* can produce collagenase and fibrinolysin, which neutralize the mucosal protective factors and facilitate adhesion to the host cells [138,139]. Likewise, Pybus and Onderdonk uncovered a special commensal relationship between *G. vaginalis* and *P. bivia* while studying how nutrient utilization contributes to the pathogenesis of BV [133]. Through a series of testing, it was then found out that *P. bivia* stimulates the growth of *G. vaginalis* by supplementing the latter with ammonia. Apart from that, the interactions between *P. bivia* and other anaerobic bacteria associated with BV, such as *Peptostreptococcus anaerobius* are worthwhile to be investigated, particularly for the assimilation of amino acids and other supplements which favors the growth of pathogenic microbes within the vaginal ecosystem [140,141].

Initially isolated as a novel strain in 1999, the facultative anaerobic *A. vaginae* was recovered from the vagina microflora of a healthy person [142]. Its role in BV has always been the center of the argument until lately, whereby researchers unveiled that the bacterium is indeed an important focal component in the abnormal microflora of BV [143,144,145,146,147,148,149]. As demonstrated through in vitro experiments, *A. vaginae* triggered the host’s innate immune response via vaginal epithelial cells, which later increased the localized production of IL-6 and IL-8 and an antimicrobial β-defensin peptide, mediated through the toll-like receptor 2 [150]. These immune responses observed were consistent with clinical features of BV; thus, researchers suggested that *A. vaginae* may possibly contribute to the pathogenesis of BV via alteration on the host immune system. A study by Ferris and team in 2004 studied the association between *A. vaginae* and BV using a molecular approach—performing 16S rRNA gene-targeted PCR and denaturing gradient gel electrophoresis (DGGE) before comparing the microbe’s presence among BV patients as well as healthy controls [144]. As predicted, only samples from patients with BV displayed *A. vaginae* amplicons with distinct band patterns in DGGE analysis, but neither amplicons nor bands appeared in healthy control samples. Another investigation done by a Belgium team revealed a high bacterial load of *A. vaginae* within the *G. vaginalis*-dominated biofilm, emphasizing the potential interactions between these two microbes [151]. An interesting article by Castro et al. explained a potential reason why *A. vaginae* rarely triggers BV alone but often in conjunction with *G. vaginalis* and its associated biofilm [149]. Nearly 92% of *A. vaginae* died within 48 h under mono-species planktonic culture conditions, but this bacterium remained viable when co-cultured with *Gardnerella* spp. or after a pre-conditioning step with cell-free supernatant of *Gardnerella* spp. cultures. There is still a lot to be studied regarding the primary roles of *A. vaginae* in BV, but accumulating evidence is showing that rather than being an “initiator” in BV pathogenesis, this anaerobe is most likely a “secondary colonizer” and symbiont of *G. vaginalis* [147,152,153]. While *G. vaginalis*-dominated biofilm provides protection and growth support upon anaerobes like *A. vaginae*, one of the reasons that urged the scientific community to investigate this bacterium thoroughly is owing to its antimicrobial resistance (AMR) pattern and strong association with recurrent BV [152,154]. Apart from displaying resistance towards metronidazole (which is a commonly used drug to treat BV), several clinical strains of *A. vaginae* also displayed resistance towards nalidixic acid and colistin [143,155,156,157]. Undeniably, the emergence of multi-drug resistant microbes has caused a substantial dilemma among the scientific community [122,154,156,158,159]. AMR can be acquired between microbes via several routes, including mutation, transduction, conjugation, and transposons; this topic has been heavily reviewed in the past 40 years or so [159,160,161,162,163,164,165]. The ability of *A. vaginae* to live and adapt within the *G. vaginalis*-dominated biofilms constitutes another threat in terms of ensuring successful treatment and complete remission of BV [166,167]. Members living within the biofilm often exchange genetic material, including those encoding for AMR and biofilm development. Using the next-generation sequencing method, Bostwick et al. performed a case-control study to study AMR genes’ prevalence within BV patients. Their results showed that AMR genes corresponding to all class of antibiotics were detected including macrolides, (35.2%), lincosamides (35.6%), tetracyclines (21.8%), aminoglycosides (streptomycin, gentamicin, and tobramycin, 5.2% each), 5-nitroimidazoles (0.3%) and triazoles (18.7%) [156]. Moreover, a significantly higher frequency of AMR genes was detected in BV pathogens as compared to healthy control (i.e., macrolides: 58.2% versus 12.3%, lincosamides: 58.9% versus 12.3%, tetracyclines, 35.6 versus 8.0%). The monitoring of AMR in BV is crucial in assisting clinicians in choosing the best treatment for patients with BV and preventing antimicrobials misuse, which can give rise to more multi-drug resistant microbes. As a matter of fact, *G. vaginalis* has recently been shown to produce membrane vesicles that are cytotoxic against vaginal epithelial cells uncovered by a team in India [168]. As membrane vesicles are proposed to be another mechanism that promotes horizontal gene transfer, including AMR genes, these findings collectively show the dire need for an effective treatment plan to treat a polymicrobial infection, as seen in BV.

## 5. Diagnostics Methods of BV

Over the years, researchers have been studying different methods and techniques to facilitate the diagnosis and identification of pathogens associated with BV. Vaginal cultures for BV diagnosis generally lack positive predictive value or diagnostic value as the infection itself usually is polymicrobial in nature [111,169,170]. Also, bearing in mind that performing conventional bacteria culture can be challenging to observe fastidious microbes and is time-consuming, resulting in misinterpretation or underdiagnosis. One of the standard methods for clinical diagnosis of BV is based on the presence of three out of four Amsel’s criteria in most clinical settings (Table 2) [171]. The four Amsel’s criteria include: (a) presence of thin grayish-white homogenous discharge, (b) vaginal pH above 4.5, (c) potassium hydroxide (KOH) test (which is also known as the positive whiff-amine test), and (d) at least 20% clue cells (which are exfoliated vaginal epithelial cells heavily coated with the less favorable microbes) can be observed on a saline wet mount [172]. Alternatively, two other methods can be used to diagnose BV—the Spiegel criteria and Nugent’s criteria. The only similarity between the three tests is the investigation of vaginal smears, though as to the interpretation process, Spiegel’s criteria and Nugent’s criteria focus on morphological observation of microbial cells without considering the presence of clue cells [173,174]. The Nugent’s criteria, also known as the Gram stain diagnosis method, is considered the gold standard for diagnosing BV based on a 10-point scale using microscopic observation of Gram-stained vaginal smears under the oil immersion method [175]. The score is given as a weighted score calculated from the average number of different morphotypes seen per oil immersion field. Usually, there are three morphotypes described: *Lactobacillus* spp. morphotype (decrease in number scored as 0 to 4), *Gardnerella* or *Bacteroides* spp. morphotype (small gram-variable rods or gram-negative rods; scored as 0 to 4), and curved Gram-variable rods (scored as 0 to 2). The BV diagnosis is confirmed with a score of ≥7; a scoring of 4–6 indicates intermediate flora, and a score of 0–3 is classified as normal flora. Essentially, the scoring system of Nugent’s criteria is derived from the Spiegel criteria, which was described in the early 1980s. However, the Nugent’s criteria present as a better, standardized scoring system with higher intercenter reliability (r = 0.82) than Spiegel criteria (r = 0.61), which led to its adoption as the gold standard for the diagnosis of BV. Additionally, the results from a study by Moussavi and Behrouzi sparked the discussion on whether Amsel’s or Nugent’s criteria are superior to another. The duo studied the sensitivity and specificity of each criterion of Amsel before comparing it to the Nugent’s criteria [176]. Using pH criteria alone showed the lowest sensitivity (61%), while the presence of vaginal discharge alone showed moderate sensitivity (63%) with the highest specificity (80%) among all four criteria when evaluated individually for BV. Equally, the examination of clue cells alone also reflected low sensitivity (67%). Altogether, these findings indicated that the use of tests in Amsel’s criteria showed lower diagnostic validity when used alone compared to Nugent’s criteria, hence further strengthening the need of fulfilling three of the four described criteria described by Amsel et al. for the diagnosis of BV [172,176,177]. The Nugent’s criteria versus Amsel’s criteria have comparable diagnostic value for BV as discussed by many research groups, with specificity and sensitivity of Amsel’s criteria reaching as high as 95.2% and 91%, respectively, when compared with Nugent’s criteria [176,177,178,179]. As the Nugent’s criteria require proper laboratory equipment and experienced technical staff, several studies highlighted that Amsel’s criteria could be used to diagnose BV to ensure patients receive the necessary treatment promptly [177,180,181,182]. At the same time, there is another simpler classification known as Hay/Ison criteria, which classified vaginal microbiome into three different categories: normal (Group 1), intermediate (Group 2), and BV (Group 3), depending on the relative amount of *Lactobacillus* morphotypes as compared to *Gardnerella* morphotypes [183]. During the first introduction of this classification, Ison and Hay successfully diagnosed BV in 83 out of 162 patients, and the method showed high sensitivity (97.5%), specificity (96%), and predictive value for a positive (94.1%) and negative (96%) test, kappa index = 0.91, when compared with the Amsel’s criteria. A few years later, Chawla et al.’s report also compared Hay/Ison classification and Nugent’s criteria [184]. Their analysis successfully diagnosed 70 BV cases (32.86%) by Nugent’s method and 87 (40.85%) BV cases by Hay/Ison classification. Based on their calculation, Hay/Ison classification’s sensitivity and specificity were determined as ≥97.2% and ≥88.1%, respectively. Collectively, these results implied the suitability of the Hay/Ison classification to be utilized as an alternative diagnosis method when there is a lack of time or expertise [183,184,185]. A team in Italy also suggested the potential of automation using the WASP^®^ automatic system (BioMérieux diagnostics) to analyze samples collected in LMB ESwab^®^ (BioMérieux diagnostics). The introduction of advanced technology helps increase reliability and shorten sampling time while ensuring timely diagnosis and treatment [186].

Besides using Amsel’s criteria as a point-of-care test, there are other commercially available diagnostic kits for BV [187,188,189]. The OSOM BV Blue test (SEKISUI Diagnostics, MA, USA) is a rapid chromogenic diagnostic kit based on bacterial enzyme activity, sialidase [187,190]. Bypassing the need for laboratory equipment and experts for the interpretation of wet mount, the detection of sialidase activity is simple–placing the vaginal swab into the BV test vessel and mixing gently before adding the developing solution as indicated by the manufacturer’s instructions [188,191]. Another optional diagnostic kit that would be used is the FemExam card (developed initially by Litmus Concepts, Santa Clara, CA, USA, now available from Cooper Surgical, Shelton, Conn) [171,192]. The kit comprises two cards: card 1 for pH and amines and card 2 for proline iminopeptidase (PIP) activity. The easy-to-understand feature makes it an attractive diagnostic kit; two swabs are provided with the kit, and each caters for one card. The vaginal fluid collected with the first cotton swab should be applied onto the pH test site and amine test within 2 min to induce a colorimetric reaction. A blue sign on each site indicates a pH of 4.7 or greater and the presence of trimethylamine. Another swab containing vaginal fluid is used on card 2 containing a *G. vaginalis* PIP activity test site. In the presence of PIP, enzymatic and colorimetric reactions would take place after rubbing the swab onto the test site containing a chromogen (Fast Red) and a PIP substrate (l-propyl-β-naphthylamide). A PIP-positive sample results in a pink color change on the swab tip within 5 min of the test. Although this kit does offer high sensitivity and specificity as compared to conventional clinical diagnosis, the FemExam two-card method comes at a high cost as West et al. estimated the cost per patient and cost per true case detected at US $8.32 and US $18.49, respectively [192,193]. Lowering the cost for these kits may improve the accessibility in developing countries or even allow its usage as home self-examination using kits, but it remains controversial due to the possible overdiagnosis [194,195].

Compared to the traditional microscopy method, molecular tools certainly offer higher sensitivity and accuracy for BV diagnosis, which can be tricky due to its polymicrobial nature [196]. Oligonucleotide probes are designed to contain a specific site that binds to a precise genomic location of a particular bacterium, and multiple probes can be used in a single reaction to detect different bacterial present in a sample. BD Affirm™ VPIII microbial identification system (Affirm, Becton, Dickinson and Company, Sparks, MD) is a commercially available kit that allows automation and can detect three organisms with a single swab, including *Candida* spp., *Gardnerella* spp., *Trichomonas* spp. [197,198]. This technique functions based on DNA hybridization and uses two distinct single-stranded probes for each microbe—a capture probe and a color development probe. Brown et al. claimed that the Affirm™ VPIII Microbial Identification Test is more sensitive than the wet mount method, as 45% of patients were tested positive for *Gardnerella* by Affirm assay compared to only 14% by the wet mount. Another Korean team compared the Affirm assay to the Nugent’s criteria as a standard diagnostic method [199]. The team concluded that the detection rates achieved in the study were not significantly different between these two methods (33.33% versus 34.8% for *Gardnerella*) and emphasized its reliability to be used as a rapid, point-of-care test. On the contrary, Dessai et al. advised careful usage as a point-of-care diagnostic tool in pregnant women as they noted a moderate sensitivity (79.8%) and a moderate specificity (80.3%) for diagnosing BV in all participants. The same article also concluded that another nucleic acid amplification test (NAAT), BD Max™ Vaginal assay (Becton Dickinson, MA, USA), showed higher diagnostic accuracy compared to the Affirm assay.

NAATs are highly sensitive methods such as PCR technologies, which can detect as low as a single copy of DNA. With the advent of more thermostable and high-fidelity PCR enzymes, more NAAT-based techniques are being introduced to the market as it enhances accuracy while improving confidence in BV diagnosis [200,201,202,203,204]. Vaginitis Plus NuSwab^®^ (LabCorp, NC, USA) is a semi-quantitative PCR analysis of the three most predictive marker organisms in BV, *A. vaginae,* BVAB-2, and *Megasphaera*-1 [205]. The total scores are calculated based on their presence: (a) score of 0 to 1 indicates negative for BV, (b) score of 3 to 6 indicates positive for BV, and (c) score of 2 indicates inconclusive results for BV. At the time of writing, limited data is available regarding the usage of this kit, which has not obtained clearance from the United States Food and Drug Administration (FDA). Apart from NuSwab^®^, there are a few more commercially available, PCR-based kits, including SureSwab^®^ BV DNA quantitative real-time PCR (Quest Diagnostic, Secaucus, NJ, USA), Aptima^®^ BV Assay (Hologic Inc., San Diego, CA, USA), Bacterial Vaginosis Panel by Real-Time PCR (Medical Diagnostic Laboratory, Hamilton Township, NJ, USA) and BD MAX™ Vaginal Panel (Becton Dickinson, Sparks, MD, USA). The Aptima^®^ BV assay has obtained clearance from the FDA in 2019 and measures three major bacterial groups: *Lactobacillus* (*L. gasseri*, *L. crispatus*, and *L. jensenii*), *G. vaginalis*, and *A. vaginae*. Coupled with the automated Panther^®^ system, the diagnosis of BV can be made based on a mathematical algorithm analysis of rRNA detection of *Lactobacillus* species, *G. vaginalis*, and *A. vaginae* [206]. Schewebke et al. have recently compared the diagnostic value of this newly FDA-cleared assay to conventional in-clinic diagnosis and clinical observations. Surprisingly, the group showed that this investigational assay reflected higher sensitivity (≥96.2%) and specificity (≥92.4%) compared to the other methods (e.g., 83.4% and 85.5% for clinicians’ diagnoses, 75.9% and 94.4% for Amsel’s criteria). As a result, the team supported the use of in vitro diagnostic NAAT, which can be automated to provide differential diagnoses of multiple etiologies from a single vaginal swab.

Founded in 1997, Medical Diagnostic Laboratory Medical Diagnostic Laboratories, L.L.C. (MDL) developed the Bacterial Vaginosis Panel by Real-Time PCR, allowing simultaneous molecular detection of five bacteria associated with BV, including *A. vaginae*, BVAB-2, *G. vaginalis,* and *Megasphaera* spp. (Type 1 and Type 2), along with four species of the *Lactobacillus* genus. [189]. A study in 2016 evaluated the selection of these nine bacteria as a potential diagnostic marker for BV via quantitation using qPCR in combination with logistic regression and receiver operator characteristic (ROC) analysis [203]. The team concluded that *G. vaginalis*, *A. vaginae*, and *Megasphaera* spp. (Type 1 and 2) provided the most accurate diagnosis of symptomatic BV based on a logistic regression model, with 92% sensitivity, 95% specificity, 94% positive predictive value, and 94% negative predictive value when compared to Amsel’s criteria and Nugent scoring. Even though the detection of *Lactobacillus* spp. did not reflect diagnostic value for BV, it may be useful as a potential prognostic marker to study the long-term sustained response to treatment or even a risk indicator for recurrent BV.

On the other hand, BD MAX™ Vaginal Panel measures BV markers along with candidiasis and *Trichomonas vaginalis* infection and can be automated on the fully integrated BD MAX™ System, which performs both nucleic acid extraction and real-time PCR. A total of five bacteria are detected, including *Lactobacillus* spp. (*L. crispatus* and *L. jensenii*), *G. vaginalis, A. vaginae*, BVAB-2, and *Megasphaera-1*. As the first paper to evaluate BD Max™ Vaginal Panel for detection service in the United Kingdom, Sherrard noted a lower sensitivity of the kit for BV at 94.4% with a specificity of 79%, compared to its previously reported value [207,208]. Nonetheless, the investigational test provides broader diagnostic coverage and is less labor-intensive than traditional tests as its automation can potentially lead to a more rapid turnaround time in the diagnostic lab.

Designed with simplicity of use and clear usage instruction, some of these molecular kits allow patients to collect the specimen themselves before sending it to the lab for analysis. Considering that molecular techniques are often very sensitive, some clinicians mention that this helps to overcome the reluctance of some women to undertake gynecological examination, originating from cultural, religious, and socioeconomic factors, while the rest remain conservative on the idea of patients’ self-collection of specimens at home [209,210,211]. Correspondingly, some clinicians raised concern about the usage of the non-FDA-approved assay(s) to detect BV and STI in children as they may produce false-positive results; at the same time, patients should be examined thoroughly before confirmation to avoid unnecessary prescription of antibiotics or related drugs [14,212].

## 6. Eradication and Management of BV-Associated Pathogens via Antibiotics

Following the discovery of penicillin by Professor Alexander Fleming in 1928, researchers actively hunted for antibiotics to treat infections in the 1950s, also known as the “golden age” of antibiotics discovery, as one-half of the commonly used antibiotics today were found during this period [213]. For BV, several international guidelines proposed two major classes of antibiotics—nitroimidazole and lincosamide antibiotics to counter the overgrowth of anaerobic microbes in the vagina (Table 3) [208,214,215,216,217,218]. The Centers for Disease Control and Prevention recommends three treatment regimens in non-pregnant patients: oral metronidazole (500 mg twice daily for seven days), intravaginal 2% clindamycin cream (one applicatorful at bedtime for seven days), or intravaginal metronidazole gel (one to two applicatorfuls per day for five days) [219,220]. Alternative regimens include a single 2 g oral dose of metronidazole or a 7-day course of oral clindamycin, 300 mg twice daily. There is no significant difference between these two antibiotics administered through oral or intravaginal routes at the time of writing, but the prescribing preference and antibiotics dosages may vary slightly in different countries. Comparing different guidelines recommending the use of vaginal metronidazole gel, data from trials indicated that there was no significant benefit for a double dose over a single dose daily, but a single dose regime may reduce non-compliance, which may ensure a higher treatment rate [221,222].

As a member of the nitroimidazole family, metronidazole constitutes a pro-drug that displays high cytotoxicity towards anaerobes and protozoa upon reduction of its nitro group [223,224]. By disrupting DNA synthesis and causing DNA damage via induction of single- and double-strand breaks, metronidazole selectively targets anaerobes (and some microaerophils like *Helicobacter pylori*) as aerobic microbes lack electron-transport proteins with sufficient negative redox potential to convert the prodrug to its active form. While some clinicians raised concern about its use in the first trimester of pregnancy and gastrointestinal side effects, this antibiotic remains the most common treatment regime for BV since its introduction in 1978 [157,225]. As described in several guidelines, clindamycin could also be used as a standard treatment for BV. Petrina et al. studied the susceptibility of BV-associated bacteria to several antibiotics, including metronidazole, clindamycin, and the newer 5-nitroimidazole, secnidazole which has a longer half-life [157]. Based on their report, metronidazole was superior to clindamycin for *Prevotella* spp., *Bacteroides* spp., *Peptoniphilus* spp., *Anaerococcus tetradius,* and *Finegoldia magna*, while clindamycin had better activity against *A. vaginae*, *G. vaginalis,* and *Mobiluncus* spp. as compared to both nitroimidazoles. At the same time, a systematic review by Oduyebo et al. showed that both clindamycin and metronidazole, which have been administered either orally or vaginally, showed similar clinical efficacy in treating BV [226]. However, those who received clindamycin showed a lower rate of adverse events than metronidazole.

As a member of lincosamides, clindamycin is a broad-spectrum antibiotic that confers bacteriostatic or even bactericidal activity at a high dose by blocking protein synthesis in susceptible bacteria via inhibition of peptidyltransferase reaction on the 50S ribosomal subunit [227,228]. To this date, it is rather difficult to pass judgment on whether clindamycin or metronidazole is better than the other as clindamycin, too, has a significant disadvantage—antibiotic resistance. In fact, one of the important factors contributing to emerging antibiotic resistance is due to the development of cross-resistance; it has been reported several times that some pathogens failed to be eradicated when treated with macrolides and lincosamides [229,230,231,232,233]. There are three key ways in which bacteria counter these antibiotics: (a) through target-site modification that prevents binding of antibiotic to its ribosomal target, (b) by increasing antibiotic efflux, and (c) by inactivating the drug. The most worrying issue is that bacteria may adopt more than one of the strategies above to ensure their survival in the environment. Beigi et al. studied the antimicrobial resistance of vaginal anaerobes in non-pregnant women with BV before and after antibiotics treatment [158]. Among the 1059 anaerobic bacterial isolates recovered from 95 women (47 metronidazole and 48 clindamycin), less than 1% demonstrated resistance to metronidazole, but the resistance towards clindamycin was appallingly high, with 17% demonstrated baseline clindamycin resistance and 53% demonstrated resistance to clindamycin after therapy. Similar findings were also reported by Austin et al. when they compared the antibiotic resistance of vaginal anaerobes after intravaginal clindamycin or metronidazole use among non-pregnant women aged 18 to 45 [234]. Vaginal *Prevotella* spp. displayed a higher level of clindamycin resistance as early as 7 to 12 days after treatment, though some were colonized initially by clindamycin-susceptible strains. Despite that, clindamycin still constitutes another treatment option for those who are allergic or intolerant to metronidazole, given that the clinical responses to BV treatment did not differ between metronidazole and clindamycin. In connection to recurrence, more than half of those treated with these antibiotics may experience BV again at the six months to one-year period following treatment [220]. A six-year observation study done by a team in Sweden revealed that 23 out of 44 women (52%) had BV relapse even after successful BV treatment using metronidazole; the team did not notice any difference in other confounding factors, such as the use of broad-spectrum antibiotics use, family planning methods, bleeding disturbances and so on between women with (mean age: 43.5 years old) and without relapse (mean age: 43.8 years old) [235]. Noting that there was no difference in hydrogen peroxide production of the *Lactobacillus* population among women with or without relapses, the team then suggested that this might be due to new sexual contacts. Complementing these results, Bradshaw et al. stated that BV recurrence might be attributed to *A. vaginae* and *G. vaginalis* population after metronidazole treatment; the proportion of women with these two microbes steadily increased over the observation period of 12 months [152]. Through these findings, both studies urged for increased awareness of antibiotics usage as clindamycin use could potentially increase the vaginal reservoir of macrolide-resistant bacteria.

Apart from metronidazole, tinidazole is a second-generation nitroimidazole with a longer half-life compared to metronidazole (12–14 h and 6–7 h, respectively) that have been proposed as an alternative BV treatment [218,225,236]. A thorough study by Armstrong and Wilson comparing the efficacy of oral tinidazole reflected variations in BV cure rates: 46% to 71% for single dose 2 g tinidazole, 74% for 2 g daily for a two-day course, and 91% for the 1 g daily for a five-day course [237,238,239,240,241]. A randomized, double-blind study conducted in India concluded that the tinidazole group (oral 500 mg once daily + one placebo for five days) had higher cure rates compared to the group receiving metronidazole (oral 500 mg twice daily for five days). The recurrence rate was observed to be lower in those receiving tinidazole than metronidazole, which might be attributed to its improved pharmacokinetic activity and a broader spectrum of antimicrobial action [242,243].

The ever-evolving of antibiotic resistance among microbes is indeed a massive challenge in infectious disease management. Dequalinium chloride has been introduced as an alternative regimen to antibiotics in the European (IUSTI/WHO) International Union against sexually transmitted infections (IUSTI) World Health Organisation (WHO) guideline on the management of vaginal discharge [218]. The mechanism of action of dequalinium chloride has been previously reviewed by Mendling et al. as they expressed that resistance is unlikely to occur due to its multiple modes of action on bacterial cell permeability and bacterial cell metabolism [244]. Despite that, some BV patients treated with dequalinium chloride may still experience adverse drug effects associated with antibiotic use, including vaginal discharge and vulvovaginal pruritus [245].

In relation to BV-affected pregnant women, the question of active screening and treatment remains a hot debate. As the protective mucus layer of the vagina degrades in BV patients, some studies observed an increased risk of acquiring and transmitting sexually transmitted diseases and calling for appropriate management plans [246,247]. While a portion of the studies reported the association of BV with other complications among pregnant women, such as an increased risk of preterm delivery, miscarriage, chorioamnionitis, postpartum endometritis, and pelvic inflammatory diseases, some studies questioned the need to treat BV in pregnant women as they do not observe a significant difference in treatment outcome.

## 7. Reinstate Balance in the Vaginal Microbiome via Probiotics Supplement

**Figure 1:**
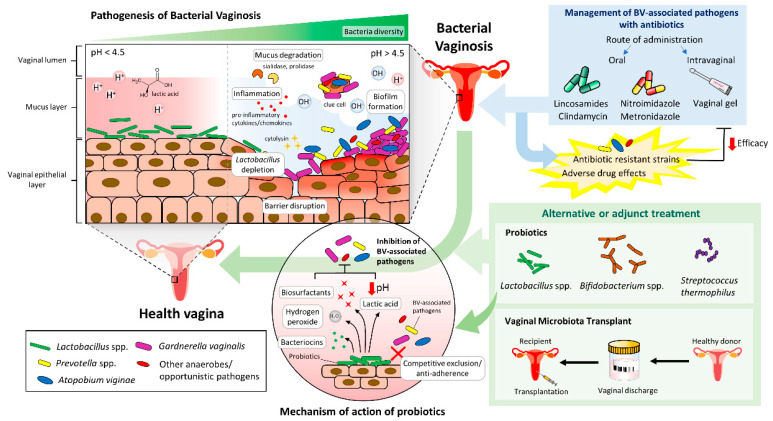
Pathogenesis of bacterial vaginosis (BV) and the mechanism of action of probiotics in battling against BV.

To reinstate the normal microflora consisting of a high abundance of *Lactobacillus* spp., researchers hypothesized that probiotics containing *Lactobacillus* spp. could be a possible choice in maintaining vaginal homeostasis (Figure 1). The general definition of probiotics is termed as live microbial feed supplements which can be taken orally or applied to have good health benefits when administered in sufficient amounts. There has been an increase in demand over the years due to the emerging evidence regarding probiotics’ potential role to modify the flora in improving several medical conditions such as acute diarrhea, eczema, and genitourinary infections [248,249]. For BV, it is hypothesized that apart from its bacteriocin production (including hydrogen peroxide) and host immunomodulatory properties, probiotics confer anti-adhesion properties via competition for the binding site, displacing the other pathogens from the adhesion site on the vaginal epithelium [104,105,250]. On top of that, some studies demonstrated that probiotics like *Lactobacillus* spp. decrease the adhesion of pathogens on the surface epithelium by producing biosurfactants with anti-biofilm activities [251]. Studying the clinical trials that describe the efficacy of probiotics against BV, patients were given either probiotics consisting of a single strain or multi-strain (i.e., a combination of *Lactobacillus* spp. and *Bifidobacterium* spp.) through oral route or inserted vaginally. On the flip side, another group of clinicians suggested using probiotics in conjunction with standard antibiotics for BV, intending to restore vaginal microflora after the bactericidal activity of antibiotics. While probiotic is still not recommended as a treatment for BV, supplementation with probiotics appears to be effective for the management of BV, regardless of concurrent antibiotics usage (Table 4, Table 5 and Table 6). More studies are still essential to understand the actual mechanism(s) involved in modulating the microbiota composition in the vagina which can eventually help clinicians to make the best decision to achieve long-term remission in BV patients.

### 7.1. Outcomes of Probiotics with Antibiotics

Most of the studies used metronidazole (500 mg twice per day) as the standard treatment for seven days, and the range of dose in probiotics was between 10^4^–10^9^ CFU (Table 4). Seven randomized controlled trials (RCTs) focused on single-strain probiotics, which are all administered intravaginally. One of them showed that *L. casei* var. *rhamnosus* (Lcr35) administered via vaginal capsule for seven days after one week of antibiotic treatment with clindamycin (300 mg twice per day) led to significant improvement (*p* < 0.001) of Nugent score assessed at five weeks in 83% women compared to 35% women in the control group [252]. Another study performed in Italy showed similar results in the probiotic group using *L. rhamnosus* once a week for two months, which reported a significant difference between the two treatment groups (*p* = 0.05) at day 90 [253]. Later in 2010, Marcone et al. recruited 49 BV patients and randomized them into two groups: one group receiving standard oral metronidazole treatment and another receiving vaginal capsule (once a week) containing 40 mg of *L. rhamnosus* for six months after discontinuing antibiotic treatment (i.e., day 8 of treatment). It is fascinating that a major portion of patients receiving *L. rhamnosus* consistently showed a balanced vaginal ecosystem in the first six months. The follow-up study over 12 months concluded that *L. rhamnosus* permitted the stabilization of vaginal microbiota and subsequently prevented BV occurrence [254].

Using *L. crispatus* IP 174178 as a probiotic, a study in Paris administered the strain intravaginally for 14 days for four menstrual cycles to women with a history of recurrent BV in the past year after seven days of standard treatment oral metronidazole. There was an increase in recurrence time by 28%, and there were a lower number of recurrent cases in the intervention group (20.6%) compared to the placebo group (41%) [73]. There were another two studies that reported the use of a different strain of *L. crispatus* in BV. Twenty-four women diagnosed with BV were recruited for a study in San Francisco, United States, and treated with 0.75% topical metronidazole for five days before sorted into the treatment and placebo arm at a 3:1 ratio. Participants in the probiotic group used LACTIN-V containing *L. crispatus* CTV-05 for five days followed by once a week for two weeks on days 12 and 19. The study further analyzed the adherence and noticed that one out of four patients who have completed all seven doses of LACTIN-V had recurrent BV. The colonization efficiency of *L. crispatus* CTV-05 evaluated using DNA fingerprint identity was found to be 61% (11/19, 95% CI: 36–83%) among all LACTIN-V users. The study also reported that only one out of 11 women colonized with *L. crispatus* CTV-05 had BV at Day 28 (9%), three of the seven participants remaining uncolonized had BV (43%). While the population size is rather small for the current study, Hemmerling et al. concluded the safe use of LACTIN-V and called for a study with a bigger sample size to confirm the effectiveness of this probiotic [255]. In the article published in 2020, Cohen et al. conducted a bigger RCT and treated BV patients with powdered Lactin-V (*L. crispatus* CTV-05) in a pre-filled vaginal applicator for 11 weeks after a five-day 0.75% metronidazole gel treatment (*n* = 152). The adherence to the assigned treatment for this study was higher than the figure reported by Hemmerling et al. at 81%, according to the participant report. In consonance with that, LACTIN-V treatment has significantly reduced recurrence of bacterial vaginosis compared to the placebo group (*p* = 0.01), as reported by Cohen et al. [256].

Besides, there are three (non-RCT) studies that investigated single strain probiotics, which were administered intravaginally. In a prospective case-control trial, standard treatment with metronidazole and *L. rhamnosus* BMX 54 restored normal vaginal flora, and the rate of BV recurrence reduced significantly as well (*p* < 0.001) [257] (Table 5). The clinical cohort trial done in China was done slightly differently as they compared seven days of vaginal metronidazole to a ten-day course of a vaginal suppository containing *L. delbrueckii,* which mentioned that the cure rate was much higher (*p* = 0.013) after 30 days in the probiotic group (96%) compared to the antibiotic group which was (70%). The postulated reason was that probiotics re-established vaginal microbiota steadily, whereas metronidazole eradicated most of the bacteria in the vagina. However, there are several biases mentioned that could have affected the study [258]. An observational study suggested that *L. plantarum* may reduce infection rate after four months, but findings were not statistically significant [259].

Among the literature studying probiotic use in combination with antibiotics, one RCT in India administered *Bacillus coagulans* Unique IS-2 as a probiotic orally in BV patients. The control group (*n* = 20) received only a course of antibiotic treatment consisting of ofloxacin–ornidazole capsule (200–500 mg/day) for five days in combination with co-kimaxazol vaginal pessaries for three days, while the probiotic group received the same antibiotic treatment together with two capsules of *B. coagulans* Unique IS-2 for 90 days. The majority of the participant receiving *B. coagulans* Unique IS-2 (16/20) were negative for white discharge and amine test compared to only nine subjects in the control group (*p* = 0.0002), suggesting a beneficial effect of the strain against BV [260].

**Table 4:** Studies reporting the use of probiotics along with antibiotics to treat BV (Randomized control trials).

Reference	Sample Size	Treatment	Mode of Treatment	Single/Multi-Strain	Probiotics Strains	Observations
[252]	190	7 days of clindamycin (2 × 300 mg) + probiotics or placebo	Vaginal capsule (1 week)	Single strain (10^9^ CFU)	*L. casei* var. *rhamnosus* (Lcr35)	Significant improvement in Nugent score at 5 weeks (*p* < 0.001)
[253]	84	7 days of oral metronidazole (2 × 500 mg/d) + probiotic or placebo	Vaginal tablet (once a week for 2 months)	Single strain (>40,000 CFU)	*L. rhamnosus*	Significant improvement (*p* = 0.05)
[254]	49	7 days of oral metronidazole (2 × 500 mg/d + probiotic	Vaginal capsule (once a week for 6 months)	Single strain (>40,000 CFU)	*L. rhamnosus*	Stabilization of vaginal microbiota up till 12 months
[73]	78	7 days of oral metronidazole (1 g/d) + probiotic or placebo	Vaginal capsule (56 days)	Single strain (10^9^ CFU)	*L. crispatus* IP 174178	Time of recurrence was longer (*p* = 0.0298) Significant reduction in recurrence (*p* = 0.0497)
[255]	24	5 days of vaginal metronidazole 0.75% gel + probiotic/placebo	Vaginal applicator once daily for 5 days followed by once weekly for 2 weeks	Single strain (10^9^ CFU)	*L. crispatus* CTV-05	Significantly lower recurrence in 28 days
[256]	228	5 days of vaginal metronidazole (0.75% gel/d) + probiotic or placebo	Vaginal (powder form; administered using prefilled vaginal applicator)	Single strain (2 × 10^9^ CFU)	*L. crispatus* CTV-05	Significantly lower recurrence of BV in 46 participants (30%) receiving probiotics compared to 34 participants (45%) in the placebo group at 12 weeks. The protective benefit persisted through week 24 in those in the probiotic group (RR 0.66; 95% CI 0.44–0.87; *p* = 0.01).
[260]	40	5 days of Ofloxacin–Ornidazole (200–500 mg) per day + 3 days of co-kimaxazol vaginal peccaries + probiotic/control	Oral capsules (90 days)	Single strain (10^9^ CFU)	*B. coagulans Unique IS-2*	Significant reduction in BV symptoms *(p* = 0.0002)
[261]	64	Tinidazole (2 g) one dose + probiotic or placebo	Oral capsule (2 capsules for 4 weeks)	Multi-strain (2 strains 10^9^ CFU)	*L. rhamnosus* GR-1, *L. reuteri* RC-14	Significant cure rate (*p* = 0.001)
[262]	578	7 days of oral metronidazole (2 × 500 mg/d) + probiotic or placebo	Oral capsule (10 days every month for 3 months)	Multi-strain (3 strains-10^8^ CFU)	*L. fermentum* 57A, *L. plantarum* 57B, *L. gasseri* 57C	Remission was lengthened significantly (p = 0.0125)
[263]	125	7 days of oral metronidazole (2 × 500 mg/d) + probiotic or placebo	Oral capsule (30 days)	Multi-strain (2 strains-10^9^ CFU)	*L. rhamnosus* GR-1, *L. reuteri* RC -14	88% were cured in probiotic group compared to 40% in placebo group (*p* < 0.001). More *Lactobacillus* spp. (>105 CFU/mL) detected on day 30 in 96% probiotic-treated subjects compared to 53% of controls
[264]	65	10 days of oral metronidazole (2 × 400 mg/d) + probiotic or placebo	Oral capsule (twice daily for 25 weeks)	Multi-strain (2 strains-2 × 10^9^ CFU)	*L. rhamnosus GR-1, L. reuteri RC-14*	No improvement in Nugent score (*p* = 0.4)
[265]	100	7 days of 2% clindamycin cream + probiotic or placebo	vaginal capsule (gelatin; 10 days for 3 cycles)	Multi-strain (2 strains-10^9^ CFU)	*L. gasseri*, *L. rhamnosus* (EcoVag^®^)	Significant improvement (*p* = 0.042) and more women were BV free after 6 months
[266]	187	3 days of vaginal clindamycin ovules (Dalacin 100 mg) + probiotic or placebo	Vaginal (tampon) (2 menstruation periods)	Multi-strain (4 strains-10^6^ CFU)	*L. fermentum*, *L. caseivar*, *L. rhamnosus,* and *L. gasseri*	No improvement in cure rate
[267]	36	7 days of oral metronidazole (2 × 500 mg/d) + twice daily probiotic or placebo	Yogurt (4 weeks)	Multi-strain (4 strains-10^7^ CFU/mL)	*L. crispatus*, *L. gasseri*, *L. rhamnosus, L. jensenii*	Significant decrease in Nugent score (*p* = 0.158) and decrease in Amsel score (*p* = 0.038)
[268]	68	7 days of oral metronidazole (2 × 500 mg/d) + probiotic Ecologic Femi (EF+)/Gynophilus LP	Vaginal capsule	Multi-strain (10^9^ CFU) EF+ (6 strains) Gynophilus LP (2 strains)	EF+ (*Bifidobacterium bifidum* W28, *Lactobacillus acidophilus* W70, *L. helveticus* W74, *L. brevis* W63, *L. plantarum* W21, *L. salivarus* W23) Gynophilus LP (*Lcr regenerans*, *L. rhamnosus* 35)	Significant reduction in BV anaerobes in EF+ (*p* = 0.043) and GynLP (*p* = 0.220)
[269]	39	Cefixime + doxycycline + metronidazole) + probiotic or no additional treatment	Vaginal capsule	Multi-strain (2 strains-10^8^ CFU)	*L. rhamnosus* DSM 14870, *L. gasseri* DSM 14869	No improvement in cure rates or recurrence
[270]	60	5 days of vaginal metronidazole (0.75% gel/d) + probiotic or no additional treatment	Oral capsule/vaginal spray (15 days: 5 days of oral capsule followed by 10 days of oral capsules along with vaginal spray)	Multi-strain (≥2 × 10^9^ CFU)	*L. acidophilus, L. rhamnosus* GG, *B. bifidum* and *B. longum*	44.8% of women cleared BV one month post-treatment. No significant differences in BV cure (RR = 0.52, 95% CI = 0.24–1.16), recurrence, vaginal pH, symptoms. Higher BV cure rates in the metronidazole only arm (63.6%; 7/11) compared to the probiotic arm (33.3%; 6/18; RR = 0.52, 95% CI = 0.24–1.16, *p* = 0.109) 1-month post-treatment. At 3-month post-treatment, half of the cured women in the metronidazole only arm (3/7) re-tested BV positive (Nugent 7–10), while more than half of the cured women in the intervention arm (4/6) remained BV negative until the end of the trial.
[271]	95	Clindamycin ovules (100 mg) ± clotrimazole vaginal tablets (200 mg) for 3 nights + probiotic/placebo	Vaginal capsule (5 days)	Multi-strain (10^8^–10^10^ CFU)	*L. gasseri* LN40, *L. fermentum* LN99, *L. casei* subsp. *rhamnosus* LN113 and *P. acidilactici* LN23	No significant difference in cure rate

**Table 5:** Other types of studies (not randomized control trials) investigating the use of probiotics for BV.

Reference	Type of Study	Sample Size	Treatment	Mode of Treatment	Probiotics Strains	Observations
[259]	Observational prospective study	124	Probiotic (+systematic antibiotic use for other bacterial infections)	Vaginal capsule (4 months)	*L. plantarum* P 17630 (10^8^ CFU)	May reduce recurrent BV infection, but findings were not statistically significant
[258]	Clinical cohort trial	121	Metronidazole/probiotic	Vaginal suppository (30 days)	*L. delbrueckii* subsp. *lactis* DM8909 (10^9^ CFU)	Significant cure rate in probiotic group at day 30 (*p* = 0.013)
[257]	Prospective case-control study	250	Metronidazole (2 × 500 mg for 7 days) + probiotic/no additional treatment	Vaginal tablet (7 months)	*L. rhamnosus* BMX 54 (NORMOGIN) (10^4^ CFU)	Significant reduction vaginal pH, symptoms, and rate of recurrence (*p* < 0.001) for 9 months

Four out of the eleven RCT studies evaluated the efficacy of using multiple strains of *Lactobacillus* spp. in treating BV via oral administration route. The probiotic mixture, which contained *L. rhamnosus* GR-1 and *L. reuteri* RC-14 was given with one dose of 2 g tinidazole twice a day for a month which showed a significant cure rate (*p* = 0.001) in 87.5% of women [261]. The clinical study in Poland incorporated three strains of *Lactobacillus*—*L. fermentum* 57A, *L. plantarum* 57B, *L. gasseri* 57C to be administered for ten days for three months, which lowered the recurrent rate significantly longer in the intervention group by 51% (*p* = 0.0125) [262]. Another clinical trial in Nigeria compared the efficacy of oral capsules containing two strains for 30 days showed positive results (*p* < 0.001) as well [263]. Another clinical trial in Africa involving HIV-infected women with a Nugent score of 4–10 also studied the use of the same combination of *Lactobacillus* spp. [264]. The study administered oral capsules containing *L. rhamnosus* GR-1 and *L. reuteri* RC-14 twice a day for 25 weeks and noticed that the treatment did not significantly improve Nugent scoring (*p* = 0.4) compared to the placebo group.

Another seven studies evaluated the efficacy of probiotics based on intravaginal application. In a study in Norway, 2% of clindamycin cream was applied for seven days followed by administration of vaginal gelatine capsule for ten days for three menstrual cycles, which included two strains resulted in significant improvement in 64.9% of women infected with BV and the majority were BV-free after six months [265]. Still, there was no improvement in terms of cure rate and recurrence in another study conducted in Africa, which incorporated three antibiotics cefixime, doxycycline, metronidazole with probiotic (two strains), or with no adjunction treatment, which was partly due to very limited power of study [269]. A study also compared the effectiveness of two different types of probiotics containing different amounts of strains [268]. EF+ contained six strains, including *Bifidobacterium* spp. which showed a significant reduction of BV anaerobes (*p* = 0.0430) when taken once for the first five days followed by thrice weekly for a duration of two months, whereas Glycophilus LP, which had two strains of *Lactobacillus* spp. also showed significant reduction compared to the control group but much lesser efficacy compared to EF+ when applied once every four days for a period of two months. Positive results were seen in women infected with BV when applying 125 g yogurt containing four strains (*L. crispatus*, *L. gasseri*, *L. rhamnosus*, *L. jensenii*) for four weeks [267]. Yet, there was no improvement in cure rate for patients that were on three days of vaginal clindamycin ovules (100 mg) followed by treatment with a tampon loaded with *Lactobacillus* (including *L. fermentum*, *L. casei* var. *rhamnosus,* and *L. gasseri*) for two menstrual periods in another study [266]. In a randomized pilot trial conducted in South Africa, there was no difference in cure rate BV (Nugent score ≤ 3) at one month among the standard of care group that applied vaginal 0.75% gel of metronidazole for five days compared to the intervention group which also applied vaginal metronidazole followed by a combination of oral probiotic capsules and vaginal spray which consisted of four different strains for a period of two weeks [270]. The study by Ehrstrom et al. reported the highest number of probiotic strains for use in an RCT studying their efficacy in BV, including *L. gasseri* LN40, *L. fermentum* LN99, *L. casei* subsp. *rhamnosus* LN113 and *P. acidilactici* LN23 [271]. Looking at the colonization pattern, no patients showed colonization of the probiotic before supplementation and 89% of BV patients (*n* = 24/27) showed colonization by one or more of the probiotic strains. However, the colonization by these probiotics appeared to drop after menstruation and only one patient displayed continuous colonization by *L. casei* subsp. *rhamnosus* LN113. Bearing in mind that a subgroup analysis was not possible as the study recruited patients with BV and/or vulvovaginal candidiasis, the study concluded that was no differences in the vaginal pH, patient ratings of vulvovaginal symptoms, or clinical cure rate between intervention and placebo group, urging for subsequent study with longer follow up period and probiotic treatment.

### 7.2. Outcomes with Probiotics Only

Hallen et al. had an early start in studying probiotics alone against BV [272]. Randomly allocated into groups, patients under the treatment arm received vaginal suppositories containing *Lactobacillus acidophilus* twice a day for six days, while those in the control group received vaginal suppositories with pure starch. An immediate follow-up after treatment completion showed a significant improvement in vaginal wet smear results; 16 out of 28 women in the probiotic group had normal findings, while none of those receiving placeboes had normal findings. Only three of the women who received the *Lactobacillus* suppository were free of bacterial vaginosis after the subsequent menstruation. Therefore, the study concluded that further studies are required to look into the colonization of *Lactobacillus* spp. after treatment to ensure a long-term outcome.

A total of seven studies investigated the usage of solely multi-strain probiotics in managing BV (Table 6). One of the considerations in prescribing multi-strains rather than single strain when studying the use of probiotics only in BV could be due to the differential activity of *Lactobacillus* spp. whereby different strains may display distinct traits like specific bacteriocin production; it would be reasonable to speculate that the use of multi-strain *Lactobacillus* spp. would grant a synergistic effect that leads to the eradication of pathogenic organisms present in the vagina, increasing colonization and confer protection to the host [259,273,274]. Out of seven studies, three studies administered probiotics in oral form. The first study was a randomized controlled cross-over study whereby participants had to consume either probiotic 1 or 2 containing three sub-strains of *L.* *crispatus*, which was given for one week with a two-week washout period [275]. Those who started with probiotics later received the placebo and vice versa. Patients who took the probiotic 2 had a significant reduction in Nugent score (*p* = 0.002). The second study administered two-strain probiotics containing *L. rhamnosus* GR-1 and *L. reuteri* RC-14 for six weeks, which resulted in significant restitution (*p* < 0.0011) towards a balanced flora in 61.5% of women [276]. The vaginal microflora was still normal in 51.1% of subjects for another additional six weeks compared to only 20.8% of participants that received a placebo. In Iran, a study revealed a significant reduction in vaginal pH after administration of oral probiotic yogurt (100 g twice a day/week) containing a mixture of four strains (*Lactobacillus bulgaris*, *Streptococcus thermophilus*, *Lactobacillus* spp., and *Bifidobacterium lactis*) compared to orally administered clindamycin (300 mg twice a day/week) (*p* < 0.0001) in pregnant women (in the third trimester) with BV [277]. Although an increase in vaginal pH is correlated with preterm birth, the study did not observe a significant difference in preterm birth, along with preterm premature rupture of the membranes and symptom-free conditions between these two groups (*p* > 0.05) [97,277]. Comparable clinical response was observed between the clindamycin group (*n* = 252/300, 84%) in comparison with the yogurt group (*n* = 241/300, 80%) without BV symptoms as evaluated through Amsel’s criteria. Hantoushzadeh et al. concluded that the oral probiotic yogurt treatment is as effective as the antibiotic treatment through their seven-day study, but a longer study period would be good to confirm the colonization of *Lactobacillus* spp. as well as the efficacy of probiotics in non-symptomatic women [277].

Four studies focused on the intravaginal administration of probiotics. In a study conducted in Italy, patients diagnosed with BV were administered vaginal tablets (Florisia™), which included three strains such as *L. brevis, L. salivarus* subsp. *salicinius,* and *L. plantarum* for one week showed Nugent score reduced remarkably (*p* < 0.05) in 61% patients compared to 19% in the control group [278]. It was also shown in another study that the administration of this vaginal tablet for eight days significantly reduced proinflammatory cytokines (IL-1β, IL-6) [279]. Women with a history of recurrent BV in China applied a vaginal capsule that had three strains: *L. rhamnosus, L. acidophilus,* and *Strep. thermophilus* for 14 days reported lower recurrent rates for 11 months [280]. Another study conducted in Italy mentioned vaginal tablets consisting of *L. fermentum* LF15 and *L. plantarum* LP01 significantly reduced Nugent score (*p* < 0.001) after four weeks, but the positive outcome was partly attributed to the presence of tara gum [281].

While most of the studies were studying the use of probiotics in reproductive-age women, an early study in 1993 by a team in Israel compared the effect of yogurt containing *L. acidophilus* (*n* = 32) among pregnant women (in their first trimester) with BV to their counterparts receiving acetic acid tampon (*n* = 32) and those without any form of treatment (*n* = 20) [282]. A next-day evaluation showed that both yogurt and acetic acid improved BV symptoms as compared to control. Even though both acetic acid and yogurt groups displayed significant improvement two months after treatment, those receiving yogurt showed earlier superior effects compared to acetic acid at one month time point post-treatment and remained free of BV symptom till the end of the study period.

**Table 6:** Studies reporting the use of probiotics only for the treatment of BV (Randomized control trial).

Reference	Sample Size	Mode of Treatment	Single/Multi-Strain (CFU)	Probiotics Strains	Observations
[272]	57	Vaginal suppository (2× daily for 6 days)	Single strain (10^8−9^ CFU)	*L. acidophilus*	Improvement in vaginal wet smear
[282]	64	Yogurt (vaginal application-2 weeks)	Single strain (10^8^ CFU/mL)	*L. acidophilus*	Significant cure rate after 1 month (*p* = 0.04)
[276]	544	Oral capsule (6 weeks)	Multi-strain (2 strains-10^9^ CFU)	*L. rhamnosus* GR-1, *L. reuteri* RC-14	Significant decrease in recurrence (*p* < 0.001)
[275]	40	Oral capsule (1 week + 2-week washout period (vice versa))	Multi-strain (3 sub-strains-10^9^ CFU/dose)	*L. crispatus*	Significant reduction in Nugent score (*p* = 0.002)
[277]	310	Oral yogurt	Multi-strain (4 strains-10^7^ CFU)	*L. acidophilus, L. bulgaris, Streptococcus thermophilus, and Bifidobacterium lactis*	Significant reduction in vaginal pH (*p* < 0.0001)
[281]	34	Vaginal tablets (4 weeks)	Multi-strain (2 strains-400 million live cells/dose)	*L. fermentum* and *L. plantarum*	Significant reduction of Nugent score in probiotic group (*p* < 0.001) in 4^th^ week
[280]	120	Vaginal capsule (14 days)	Multi-strain (3 strains–8 billion CFU)	*L. rhamnosus*, *L. acidophilus,* and *Strep. thermophilus*	Lower recurrence rates for 11 months after treatment (*p* < 0.001)
[279]	159	Vaginal tablet (8 days)	Multi-strain (3 strains-10^9^ CFU)	*L. brevis* (CD2), *L. salivarius* subsp. *salicinius* (FV2), *L. plantarum* (FV9)	Significant reduction in (*IL*-1β, IL-6) pro-inflammatory cytokines (*p* < 0.001)
[278]	39	Vaginal tablet (1 week)	Multi-strain (3 strains-10^9^ CFU)	*L. brevis* CD2, *L. salivarius* subsp. *salicinius* (FV2), *and L. plantarum* (FV9)	Significant reduction of symptoms and recurrence (*p* < 0.05)

## 8. Can Prebiotics or Synbiotics Help to Relieve BV as Well?

Prebiotics are termed as non-viable compounds that confer health benefits by supporting the growth of microbiota, which seems attractive as an alternative to probiotics or alongside (as symbiotic) in the management of BV [283,284]. Way back in 1998, the clinical trial in Turkey evaluated the efficacy of a synbiotic containing probiotic *L. acidophilus* BV along with estriol and lactose [285]. The study has recruited a total of 307 subjects divided into three groups: (a) group I (*n* = 114) receiving only standard oral metronidazole treatment, (b) group II (*n* = 96) receiving the standard oral metronidazole treatment and vaginal suppository containing lyophilized *Lactobacillus acidophilus*, 0.03mg estriol, and 600 mg lactose for twelve days, (c) group III (*n* = 97) were only treated with only probiotics with similar ingredients and period to group II. The cure rate for group I (87.7%) and II (92.7%) are similar but was significantly higher than the third group (55.6%) (*p* = 0.0001). These observations raised doubts on the efficacy of probiotics alone against BV, but the same study reported significantly higher vulvovaginal candidiasis in group I (12.2%) compared to group II (3.1%) and III (2.1%). Consequently, the study concluded that the treatment plan of standard antibiotic treatment in combination with estriol and lactose is the best in terms of cure rates and secondary vulvovaginal candidiasis rates.

A study in 2012 by Coste et al. indicated better recovery towards a normal vaginal flora in women using prebiotic gel with APP-14 for 16 days (containing gluco-oligosaccharides that encourage selective growth of several beneficial *Lactobacillus* spp.) [286]. The protective effect of prebiotic against BV is inspiring given that only two patients in the group receiving prebiotic (*n* = 2/9, 11%) reported a new BV episode on day 84 compared to four patients in the placebo group (*n* = 4/21, 19%). Even though the recurrence between these two groups was statistically insignificant (due to the small sample size), the group highlighted that prebiotic could be an exciting method to facilitate the growth of the *Lactobacillus* population in situations where they have been depleted, including BV. Comparable comments were also noted from a team in Iran, as Hakimi et al. discovered that the cure rate was significantly higher in groups that used prebiotic (Trifolium vag) as an adjuvant treatment with oral metronidazole (76%) compared to the control group (30%) on day 10 (OR: 4.3; 95% CI: 2.7–9.4) at day 10 and day 90 after treatment [287]. Both studies described well tolerability and high satisfaction by women receiving prebiotic gel, which further upholds their potential to be evaluated in clinical studies with a larger cohort to be used as a treatment option for BV.

The benefits of glycoprotein lactoferrin have been implicated in numerous literatures, conferring multiple bioactivities, including antimicrobial, anti-inflammatory, and anticancer [288,289,290,291]. Among healthy women, the probiotic mixture, Respecta^®^ consisting of *L. rhamnosus* HN001 (L1), *L. acidophilus* La-14 (L2), and lactoferrin RCXTM has successfully increased their abundance in the vagina when taken orally [292]. While no conclusion can be drawn by this study on therapeutic potential, Jang et al. adapted the protocol in a BV animal study and noticed that both oral and intravaginal administration of Respecta^®^ attenuated *G. vaginalis*-induced BV with decreased epithelial cell disruption and myeloperoxidase activity [292,293]. Subsequently, Russo et al. in Romania recruited 48 adult women to investigate the efficacy of Respecta^®^ given as an adjuvant with oral metronidazole treatment [294]. A longitudinal study showed that oral metronidazole and placebo improved vaginal discharge in the first week, but the condition recurred after that. The combination of Respecta^®^ and metronidazole decreased vaginal discharge since the first week, specifically 87.5% and 70.8% of the participants showed significant improvement at 4-month and 6-month time point after completion of treatment as compared to the placebo group (37.5% and 33.3%, *p* < 0.05). Additionally, participants receiving Respecta^®^ reflected an improvement in Nugent score and reduction of recurrence rate, suggesting that a bigger RCT may help develop this strategy as a safe, effective alternative for the management of BV.

## 9. Vaginal Microbiota Transplant: A More Innovative Approach?

Aside from the conventional treatment using drugs, another exciting approach that has been proposed not long ago to treat human diseases is microbiome transplants. Fecal microbiota transplant (FMT) has been under the limelight since its success in treating recurrent *Clostridioides difficile* infection. Since then, researchers have begun to investigate its potential therapeutic effects in diseases including inflammatory bowel disease, obesity, diabetes, liver diseases, or even colorectal cancer [295,296,297,298,299,300,301]. Inspired by FMT, vaginal microbiota transplant (VMT) is the next “in-trend” topic, whereby researchers intend to “reset” the vaginal microbiome to a healthy state by implanting vaginal discharge from a healthy individual to the recipient(s) with BV [302,303]. Based on records retrieved from ClinicalTrials.gov (which is maintained by the United States National Library of Medicine at the National Institutes of Health), there are three ongoing studies involving the intervention of VMT. One of the studies has recently published their findings, suggesting the immense potential of VMT to be used to treat BV (ClinicalTrials.gov identifier: NCT02236429) [303]. In the report, Lev-Sagie et al. recruited a total of five patients with recurrent BV for VMT procedures; four of them eventually showed improvements of symptoms and were associated with full long-term remission until the end of the follow-up (at 5 to 21 months after VMT). Apart from improvements observed based on Amsel’s criteria, the team also studied the vaginal microbiome using 16S rRNA and shotgun metagenomics approach on the Illumina MiSeq and Illumina NextSeq platform, respectively. It was not surprising that the vaginal microbiome of a healthy donor and BV patients clustered differently in the principal-coordinates analysis. Notably, these patients displayed a rapid change in bacterial composition as early as one-month post-VMT (which correlated with recovery in Amsel’s criteria) and became significantly more like their respective donor’s vaginal microbiome configuration. Being the first exploratory study testing the use of VMT for recurrent BV, the same study pointed out that patients may need more than one VMT session to achieve remission. One of the patients initially reported partial symptomatic improvement after the first VMT session along with negative Amsel’s criteria and normal microscopic findings for four weeks of follow-up; nonetheless, her symptoms came back, and she was tested positive based on Amsel’s criteria after taking a course of antibiotics for pharyngitis. Still, the second session of VMT partially improved her symptoms and led to normalization of all Amsel’s criteria for 6.5 months of follow-up.

Regarding safety concerns associated with the procedure, the team did not observe any adverse effects associated with VMT, which subsequently emphasized the potential of VMT in treating BV, particularly for women suffering from recurrent, intractable BV and failed to respond to antibiotics. Despite that, it may be too early to exclude the risk of contracting infectious agents when proceeding with microbiota transplantation. Previously, FDA had issued warnings regarding the investigational use of FMT for recurrent *C. difficile* when patients contracted enteropathogenic *Escherichia coli* (EPEC) and shigatoxin-producing *Escherichia coli* (*n* = 2) during the procedure; the agency subsequently suggested the need to inform patients about the potential risk of pathogenic bacteria [304,305]. Consequently, Lev-Sagie et al. incorporated a list of stringent criteria for VMT donor selection, including no history of BV in the past five years, free of sexually transmitted infection, consumption of herbal or homeopathic remedies, and so on [303]. The team specifically highlighted the probable transfer of infectious agent(s), along with the establishment of a “vaginal fluid bank” as a centralized source that performs routine quality checks on donors and their specimens. Weighing out the pros and the cons, VMT presents an innovative method to re-introduce balance in the vaginal microbiota, especially for those who displayed persistent, recurrent BV that failed to respond to treatment. Perhaps, with an improved diagnostic tool and detection methods to decrease the risk of pathogenic organisms’ transfer, more in-depth studies on the efficacy of VMT in treating BV could accelerate its development as a treatment option to achieve long-term remission.

## 10. Conclusions and Future Recommendations

Although the management of BV using standard antibiotics seems to be useful for some patients, there are still many women reporting BV recurrence and dissatisfaction after treatment. Sexual behavior has been strongly implicated in the pathogenesis of BV, which then sparked another idea among clinicians—perhaps treating affected women’s sexual partners with antibiotics could reduce the occurrence and recurrence of BV? Unfortunately, the etiology of BV is still a mystery, and researchers are split on the necessity to prescribe antibiotics to sexual partners of women with bacterial vaginosis as studies are still showing mixed results [306,307]. Hence, the usage of antibiotics does not seem to be a long-term solution, given that the emergence of multidrug resistance microbes is on the rise.

In this review, probiotics, which mainly comprises *Lactobacillus* spp. seem to be a beneficial management plan as they help to reinstate balance in the vaginal microbiome. Reid et al. attempted to explore the benefits of two common inhabitants of the female urogenital tract, *L. rhamnosus* GR-1 and *L. reuteri* RC14, as probiotics in vaginal health [308]. The team noticed a significant increase in vaginal *Lactobacillus* at day 28 and 60, accompanied by a significant depletion in yeast and a significant reduction in coliforms for women treated with the probiotics when compared to control (age range: 19–46). Their results demonstrated that this probiotic mixture is not only safe for daily use in healthy women but also prevents vaginal colonization by potentially pathogenic bacteria and yeast. To date, probiotics are still not listed as a recommended treatment as a Cochrane Review published in 2009 highlighted the lack of evidence in favor or against its recommendation in the treatment of BV [309,310]. As mentioned by Senok et al., the major challenges in comparing the outcome of different trials are the variation in study design and probiotic preparations [309,311]. Even though no adverse effects were reported with probiotics administration as opposed to antibiotic use in BV, higher-quality studies are required to validate its efficacy. From the gathered literature, administration of high doses probiotics of at least 10^9^ CFU and using a probiotics cocktail may confer greater benefits than a single strain probiotic. Besides optimizing the dose and number of strains included in the probiotic formulation, the administration route is also deemed to be another critical factor when designing the treatment plan for BV. So far, clinical studies conducted to study the efficacy of probiotics in BV were delivered either orally or intravaginally. Over these years, it was observed that oral probiotics could be passed to the urogenital and vaginal system from the gut through unknown mechanisms [292,312]. However, the probiotics’ efficacy could be affected based on the ability to survive the harsh environment in the gastrointestinal system, and it would take much longer to reach the vagina. In addition to that, probiotics could be considered as an adjunct to antibiotic treatment, but more evidence is required before it is recommended. There is also another possibility to combine the use of probiotics with prebiotics as synbiotic to increase colonization rate, preventing biofilm formation, and restore the normal flora in the vagina.

Gaining attention from experts in bioscience and medical fields, the triumphant story of FMT in treating recurrent *C. difficile* has motivated researchers to attempt restoring vaginal health using VMT [313]. Limited results are available now to back the “true” efficacy of VMT, which leads to the speculation that the adoption of VMT into standard treatment for BV in the nearest future is highly unlikely. Working hand in hand with current clinical diagnostic standards, molecular tools are highly sensitive in assisting the identification and monitoring of causative agent(s), especially for the surveillance of antibiotic resistance genes [314,315]. The donor’s screening process for VMT should also be carefully planned to prevent the accidental transfer of pathogenic organisms, including HIV, human simplex viruses, or even human papillomaviruses. Anyway, the fight against the COVID-19 pandemic is still ongoing. Likewise, questions raised by experts on andrology, researchers are still on the lookout for severe acute respiratory syndrome coronavirus 2 (SARS-CoV-2) in the FRT and possible impact on vaginal health [316,317,318,319,320,321,322]. Currently, there is no substantial evidence suggesting possible transmission of SARS-CoV-2 through sexual contact, but it is still critical to detect and observe its presence in donors for VMT until a better understanding of SARS-CoV-2 is obtained. The recovery road in BV may not be as straightforward as it appears due to the complexity of the infection and patient’s risk behaviors; the management of BV is possible with more comprehensive studies shedding light on different approaches, specifically deploying a microbiome-based strategy like VMT or probiotics, prebiotic or synbiotic supplements (as adjuvant or treatment) regimen.

## Figures and Tables

**Table 1:** Prevalence of bacterial vaginosis (BV) in different countries (N/S, not stated).

Study	Country	Study Period	Age Range (Years Old)	Diagnostic Process	Population Characteristics	BV Prevalence		
						Overall (95% CI or Proportion)	Based on Age (95% CI or Proportion)	Based on Race/Ethnicity
[76]	United States	2001–2002 2003–2004	14–49	Self-collected vaginal swabs and Nugent’s criteria	Female participants selected from the National Health and Nutrition Examination Survey study	29.2 (27.2–31.3)	14–19 years old: 23.3 (20.1–26.9) 20–29 years old: 31.2 (27.0–35.6) 30–39 years old: 28.8 (25.4–31.1) 40–49 years old: 31.3 (27.3–35.6)	White/non-Hispanic: 23.2 (20.8–25.8) Black/non-Hispanic: 51.6 (47.9–55.2) Mexican American: 32.1 (27.7–36.9) Other race/Hispanic: 36.6 (27.6–45.9)
[71]		2001–2004	14–49	Self-collected vaginal swabs and Nugent’s criteria	Female participants selected from the National Health and Nutrition Examination Survey study (80.5% of total interviewed or 83.6% of those examined)	29.2 (27.2–31.3)	14–19 years old: 23.3 (20.1–26.9) 20–29 years old: 31.1 (27.1–35.7) 30–39 years old: 28.1 (25.4–31.1) 40–49 years old: 31.3 (27.4–35.7)	White/non-Hispanic: 23.2 (20.8–25.9) Black/non-Hispanic: 51.4 (47.9–55.2) Mexican American: 31.9 (27.6–36.9) Other race/Hispanic: 25.9 (19.3–34.8)
[77]	Mexico	January–July 1994	Not available	Nugent’s criteria	Patients attended to the City Hospital for a regular gynecological consultation	16.5 (67/405)	N/S	N/S
[78]	Grenada	January 2009–December 2011	15–49	Pap smears	Women participated in the national centralized data bank at the General Hospital in St George’s, Grenada	19.5	20–29 years old: 43.6%	N/S
[79]	Peru	December 1997–June 1998	18–67	Vaginal swab collection at clinic, Amsel’s criteria, and Nugent’s criteria	Women registered through community-based organization in the ReproSalud Project (promote the improvement of reproductive health)	Amsel’s criteria: 33.6 (251/748) Nugent’s criteria: 40.8 (305/748) Either criteria: 43.7 (327/748) Both criteria: 30.6 (229/748)	>25 years old: 45.5 (275/604) ≤25 years old: 36.1 (52/144)	N/S
[80]		April–August 2001	18–30	Self-collected vaginal swabs, sialidase, and PCR tests	Women recruited in a cross-sectional, population-based study to investigate the prevalence and the risk factors for HIV and other STDs	26.6 (207/779)	N/S	N/S
[81]	United Kingdom	January 1996–September 1998	<45	Amsel’s criteria	Women patients presenting for diagnosis at a genitourinary medicine clinic in Sheffield	12.9 (1161/8989)	N/S	N/S
[82]		N/S	15–49	Spiegel’s criteria	Pregnant women (gestational ages of 15 and 24 weeks) attended the antenatal clinic at Barnsley District General Hospital, Barnsley	3.54 (38/1073)	11–20 years old: 3.23 (6/186) 21–30 years old: 4.32 (30/695) 31–35 years old: 1.48 (2/135) 36–40 years old: 0 (0/44) >40 years old: 0 (0/13)	N/S
[83]	India	March–June 2002	18–40	Nugent’s criteria	Women living in slum areas of Chennai recruited for HIV intervention study	25 (120/487)	N/S	N/S
[84]		September–December 2002	15–49	Vaginal swab at health centers and Nugent’s criteria	Community-based stud	32.8 (26.38–39.22)	15–24 years old: 34.2 (13/38) 25–49 years old: 32.5 (57/175)	N/S
[85]		June–December 2004	18–40	Nugent’s criteria	Women sex workers in Chennai recruited for HIV intervention study	45 (40.6–48.7)	N/S	N/S
[86]		December 2016–November 2018	42–64	Vaginal swab at health centers and Nugent’s criteria	Outpatient female attended obstetrical and gynecological at Department of Obstetrics and Gynecology of Veer Surendra Sai Institute of Medical Sciences and Research, Burla, Odisha, India	9.1 (19/209)	≤55 years old: 43.1 (22/120) ≥55 years old: 56.9 (29/89)	N/S
[87]	Nepal	November 2014–May 2015	10–60	Nugent’s criteria	Women attended KIST Medical College Teaching Hospital	24.4 (39/160)	10–20 years old: 1.3 (2/7) 20–40 years old: 8.1 (13/48) 30–40 years old: 8.8 (14/75) 40–50 years old: 5 (8/22) 50–60 years old: 1.3 (2/8)	N/S
[88]	Ethiopia	November 2011–April 2012	18–40	Vaginal swab collection at clinic and Nugent’s criteria	Outpatient female visited the obstetrical and gynecological clinical for antenatal care services of a tertiary level hospital in Addis Ababa	19.4 (49/183)	≤20 years old: 17.6 (3/17) 21–29 years old: 21.2 (33/156) ≥30 years old: 16.5 (13/79)	N/S
[89]		September 2015–July 2016	15–64	Vaginal swab collection at clinic and Nugent’s criteria	Women attended gynecology and antenatal clinics	48.6 (102/210)	15–24 years old: 41.5 (22/53) 25–44 years old: 47.8 (56/117) 45–64 years old: 60 (24/40)	N/S
[90]	Nigeria	7 December 2010–15 July 2011	15–42	Vaginal swab collection at clinic and Amsel’s criteria	Pregnant women presenting to the antenatal clinics of the University of Maiduguri Teaching Hospital	17.3 (69/400)	15–19 years old: 23.6 (25/106) 20–24 years old: 17.5 (32/183) 25–29 years old: 16.1 (10/62) 30–34 years old: 15 (6/40) 35–39 years old: 50 (3/6) 40–44 years old: (1/3)	
[91]		November 2011–March 2012	19–45	Vaginal swab collection at clinic and Nugent’s criteria	Non-pregnant asymptomatic women attended the gynecological clinic	40.1 (85/212)	<20 years old: 100 (1/1) 20–24 years old: 43.8 (7/16) 25–29 years old: 35.5 (22/62) 30–34 years old: 41.7 (28/67) 35–39 years old: 40.5 (15/37) ≥40 years old: 37.5 (12/32)	N/S
[92]	Iran	2006	18–40	N/S	Pregnant women (before 20th week of gestation) registered in health centers	17.5 (136/777)	<30 years old: 32.7 (124/379) >30 years old: 32 (39/122)	N/S
[93]	Albania	1 January 2010–31 December 2010	N/S	Vaginal swab at clinic and Amsel’s criteria	Pregnant women attended to the Regional Hospital of Fier	23.3 (35/150)	N/S	N/S
[94]	Italy	April 1998–April 2001	40–79	Vaginal swab at clinic and Nugent’s criteria	Non-pregnant women visited three clinics in northern Italy for routine gynecologic examinations	7.6 (113/1486)	N/S	N/S

**Table 2:** Comparison between Amsel’s, Nugent’s, and Hay/Ison criteria (BV: bacterial vaginosis) (Adapted from Hainer and Gibson [171]).

	Amsel’s Criteria	Nugent’s Criteria *	Hay/Ison Criteria
Type	Clinical and laboratory diagnosis	Laboratory diagnosis	Laboratory diagnosis
Diagnosis duration	Fast	Long	Long
Expertise requirement	Clinicians	Experienced laboratory technicians and pathologist	Experienced laboratory technicians
Laboratory requirement	Low	High	Moderate
Grading system	Diagnosis is confirmed when three out of four criteria are fulfilled. (a) presence of thin grayish-white homogenous discharge (b) vaginal pH > 4.5 (c) potassium hydroxide (KOH) or the positive whiff-amine test (d) at least 20% of clue cells observed on a saline wet mount	Score 0–4: Normal flora Score 4–6: Intermediate Score ≥ 7: BV	Group 1: Normal flora (*Lactobacillus* only) Group 2: Intermediate (*Lactobacillus* = *Gardnerella*) Group 3: BV (*Lactobacillus < Gardnerella*)

* *Lactobacillus* spp. morphotype (decrease in number scored as 0 to 4), *Gardnerella* or *Bacteroides* spp. morphotype (small gram-variable rods or gram-negative rods; scored as 0 to 4), and curved Gram-variable rods (scored as 0 to 2).

**Table 3:** Treatment guidelines for non-pregnant women with bacterial vaginosis.

Guidelines	Regime Type	Drug (Dose), Frequency and Duration
Guidelines for the Management of Sexually Transmitted Infections [214]	Standard	Oral metronidazole (400 or 500 mg), twice daily for 7 days
	Alternative	Oral metronidazole (2 g), single dose
		Intravaginal clindamycin cream (2%, 5 g), once daily for 7 days
		Intravaginal metronidazole gel (0.75%, 5 g), twice daily for 5 days
		Oral clindamycin (300 mg), twice daily for 7 days
UK National Guideline for the management of Bacterial Vaginosis [215]	Standard	Oral metronidazole (400 mg), twice daily for 5–7 days
		Oral metronidazole (2 g), single dose
	Alternative	Intravaginal metronidazole gel (0.75%), once daily for 5 days, OR
		Intravaginal clindamycin cream (2%), once daily for 7 days
		Oral tinidazole (2 g), single dose
Sexually Transmitted Diseases Treatment Guidelines [216]	Standard	Oral metronidazole (500 mg), twice daily for 7 days
		Intravaginal metronidazole gel (0.75%, 5 g), once daily for 5 days
	Alternative	Intravaginal clindamycin cream (2%, 5 g), once daily for 7 days
		Oral tinidazole (2 g), once daily for 2 days
		Oral tinidazole (1 g), once daily for 5 days
		Oral clindamycin (300 mg), twice daily for 7 days
		Intravaginal clindamycin ovules (100 mg), once daily for 3 days
Australian STI Management guidelines for use in primary care [217]	Standard	Oral metronidazole (400 mg), twice daily for 7 days
		Intravaginal metronidazole gel (0.75%, 5g), once daily for 5 days
	Alternative	Intravaginal clindamycin cream (2%), 5 g once daily for 7 days
		Oral clindamycin (300 mg), twice daily for 7 days
		Oral metronidazole (2 g), single dose
		Oral tinidazole (2 g), single dose
European International Union against sexually transmitted infections (IUSTI) World Health Organisation (WHO) guideline on the management of vaginal discharge [208,218]	Standard	Oral metronidazole (400–500 mg), twice daily for 5–7 days
		Intravaginal metronidazole gel (0.75%), intravaginally, once daily for 5 days
		Intravaginal clindamycin cream (2%), once daily for 7 days
	Alternative	Oral metronidazole (2 g), single dose
		Oral tinidazole (2 g), single dose
		Oral tinidazole (1 g) orally, once daily for 5 days
		Oral metronidazole (300 mg), twice daily, for 7 days
		Intravaginal dequalinium chloride tablet (10 mg), once daily for 6 days
